# Multi-Objective Optimization of Microalgae Metabolism: An Evolutive Algorithm Based on FBA

**DOI:** 10.3390/metabo12070603

**Published:** 2022-06-29

**Authors:** Monica Fabiola Briones-Baez, Luciano Aguilera-Vazquez, Nelson Rangel-Valdez, Ana Lidia Martinez-Salazar, Cristal Zuñiga

**Affiliations:** 1 TECNM/Instituto Tecnológico de Ciudad Madero, División de Estudios de Posgrado e Investigación, Los Mangos 89440, Mexico; D00070469@cdmadero.tecnm.mx (M.F.B.-B.); luciano.av@cdmadero.tecnm.mx (L.A.-V.); 2 CONACyT—TECNM/Instituto Tecnológico de Ciudad Madero, División de Estudios de Posgrado e Investigación, Los Mangos 89440, Mexico; nelson.rv@cdmadero.tecnm.mx; 3 Department of Biology, San Diego State University, 5500 Campanile Drive, San Diego, CA 92182, USA; czuniga2@sdsu.edu

**Keywords:** cell metabolism, FBA, multi-objective optimization, nsgaii

## Abstract

Studies enabled by metabolic models of different species of microalgae have become significant since they allow us to understand changes in their metabolism and physiological stages. The most used method to study cell metabolism is FBA, which commonly focuses on optimizing a single objective function. However, recent studies have brought attention to the exploration of simultaneous optimization of multiple objectives. Such strategies have found application in optimizing biomass and several other bioproducts of interest; they usually use approaches such as multi-level models or enumerations schemes. This work proposes an alternative in silico multiobjective model based on an evolutionary algorithm that offers a broader approximation of the Pareto frontier, allowing a better angle for decision making in metabolic engineering. The proposed strategy is validated on a reduced metabolic network of the microalgae *Chlamydomonas reinhardtii* while optimizing for the production of protein, carbohydrates, and CO${}_{2}$ uptake. The results from the conducted experimental design show a favorable difference in the number of solutions achieved compared to a classic tool solving FBA.

## 1. Introduction

Microalgae are unicellular photosynthetic organisms. They are capable of capturing gases such as CO${}_{2}$ from internal combustion engines and industries, and converting it into oxygen [1]. Furthermore, some strains of microalgae have the ability to thrive under stress conditions while removing oxygen peroxide, nitrates, and phosphates present in wastewater [2], making microalgae suitable for several bioremediation strategies. In addition, microalgae CO${}_{2}$ capture through photosynthesis and its transformation into several industrial raw materials such as carbohydrates, lipids, proteins, pigments, aromatic compounds, etc., is a more economical and attractive renewable source [3,4].

A large number of strains of microalgae have been studied, finding several metabolic pathways involved in the synthesis of many secondary metabolites. However, the production rate of these metabolites varies from one species to another or even in the same species, according to different environmental and metabolic conditions. The production of the secondary metabolites depends on many factors, such as the type of microalgae and the culture conditions, temperature, pH, lighting, and nutrient sources [5].

So far, metabolic models built from genomic sequences allow a quantitative view of the transport and metabolism of compounds within a target organism. In addition, these metabolic models have long been used to generate optimized design strategies for an improved production process [6].

Most metabolic models of microalgae focus on studying a single objective function, e.g., biomass. For the particular case of metabolic networks in steady-state, Flux Balance Analysis (or FBA) is the most commonly used optimization method for their study [7,8]. Equation (1) defines the associated FBA linear optimization problem [8], where *v* is the flux vector across the reactions. The stoichiometric matrix $S_{m\times n}$ represents the metabolic network, where there is a metabolite per row and a reaction per column. The value of the cell $S_{ij}$ is the stoichiometric coefficient of the metabolite *i* involved in reaction *j* [7], and the $LB_{j},UB_{j}$ are the lower and upper bounds for the fluxes allowed in the metabolic system. The steady-state assumption is established by Sv=0 [9].

(1) FBAmaxF(v)=vbiomassSubjecttoS·v=0LBj≤vj≤UBj,∀j∈{1,⋯,n}

The solution space for FBA is defined by Equation (1), and within it optimizes a single bioproduct of interest, usually biomass. Such is the case of the application of FBA on photosynthetic organisms models, including *Synechocystis* sp. PCC 6803 [10,11], *Synechococcus* sp. PCC 7002 [12,13], *Cyanothece* sp. ATCC 51142 [14], *C. reinhardtii* [15], *Anabaena* sp. *UTEX 2576* [16], *Chlorella vulgaris UTEX 395* [6], *Chlorella variabilis* [17], *Chlorella protothecoides* [18], and *Arabidopsis thaliana* [19] to estimate fluxes and yields.

However, despite the widespread use of FBA to predict fluxes in large-scale networks, it is not always accurate in predicting fluxes in vivo [20]. Moreover, most metabolic models satisfy n>m, meaning that multiple solutions might be found. This situation becomes more complex in simultaneous bioproducts optimization. A recent trend that works in metabolic analysis involves optimizing several objectives to engage in the study of more than one bioproduct of interest [21,22,23]. In the past decade, this method can be traced back to the work of Zomorrodi and Maranas [24]. There, they developed the computational framework OptCom for FBA of microbial communities. The foundation of the framework is multi-level optimization; it optimizes problems embedded one another in a hierarchical structure for the sake of reaching optimum values for the final chosen bioproduct. Budinich et al. [21] extend FBA for microbial communities by defining a Multi-Objective FBA (MOFBA) in order to study multiple trade-offs between nutrients and growth rates. More recently, Andrade et al. [22] and Pelt-KleinJan [23] proposes a multi-objective formulation of FBA that considers nutrient limitations for metabolic analysis.

Multi-objective optimization has been exploited in a wide variety of fields in science and engineering [25,26]. MOFBA, in particular, appears in medicine, where Zhang and Boley [27] proposed a non-linear MOFBA to explain the impact of the objectives cells in the Warburg effect in different cell types. Moreover, the works [21,24,27] simulate genome-scale metabolic models for microbial ecosystems as a single strain exchanging; they use multi-objective flux equilibrium analysis, and flux variability analysis (MO-FVA).

**Figure 1:**
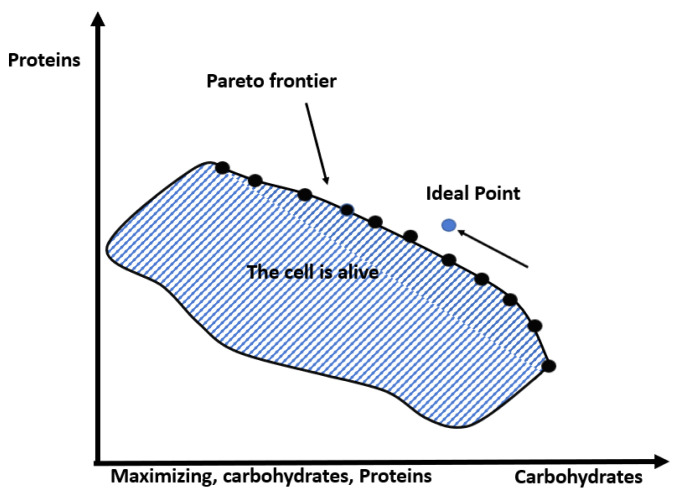
Two-objective Pareto frontier.

The main goal for multi-objective optimization is a good approximation of the Pareto frontier, cf. [28]; Figure 1 illustrates this within a metabolite context in a bi-objective function maximizing carbohydrates and proteins [29]. In this field, Multi-objective Evolutionary Algorithms (MOEAs) are widely recognized. Mainly, the algorithm NSGAII (Non-Dominated Sorting-based multi-objective EA) proposed by [30,31] has been quite effective when handling two or three objectives [32,33]. Based on the survey in [34], the only related work that uses NSGAII for FBA optimization is by Costanza et al. [35].

An overall view of the previous analysis indicates that the motivation for using multi-objective optimization in FBA lies in improving the prediction capability of FBA. However, the revised approaches do not adequately exploit the versatility of metaheuristics to approximate the Pareto frontier under a moderate consumption of computational resources. In other words, using a metaheuristic can better approximate the Pareto frontier, and provide a greater diversity of solutions than the previous approaches [25,26].

Hence, this work proposes a novel implementation of the metaheuristic algorithm NSGAII [30] for microalgae growth optimization. The novelty in the proposed NSGAII includes an original encoding scheme or genotype and an original fitness evaluation function. While in [35], NSGAII uses a knockout vector as genotype or encoding scheme, and *OptKnock* (cf. [36]) as fitness evaluation, the proposed NSGAII uses an original encoding scheme that generalizes the previous one, and an original fitness function evaluation based on FBA. The proposed encoding scheme is a generalization because the associated solutions’ search space includes the knockouts. The use of FBA instead of *OptKnock* as a fitness function might significantly impact the performance of the algorithm because instead of solving a costly combinatorial optimization problem as in *OptKnock*, it solves a simpler linear equation system.

The conducted experimental design demonstrates the validity against a glyclolysis module of a reduced metabolic network for microalgae *Chlamydomonas reinhardtii* [20]. Moreover, the proposed NSGAII is compared against FBA, and the results show that while the quality of the solution remains, the proximity to an ideal point is improved statistically and it achieved a greater diversity of solutions. Hence, the main contributions are the novel multi-objective optimization problem for metabolic analysis and the metaheuristic algorithm to solve it.

## 2. Results

Table 1 summarizes the performance of NSGAII and FBA. Column 1 shows each configuration considered. Column 2 shows the quality of solution Q achieved by NSGAII. Columns 3 to 5 show the value of Q for FBA considering as objectives each of the bioproducts chosen in the associated configuration. Finally, Column 6 presents the number of solutions produced by NSGAII; this number denoted $F_{0}$, is the number of non-dominated solutions reported by the algorithm.

The solution quality was statistically compared between NSGAII and the distinct solutions reported by FBA for each configuration, and each objective took the lead. The null hypothesis $H_{0}$ was tested: the medians of the differences between the two group samples are equal. Using the Wilcoxon statistical test with a significance level set to 0.01, and as pairs of group samples $\left(\mathrm{NSGAI}I_{\mathrm{Min}\mathrm{Euclid}},\mathrm{FB}A_{Obj_{1}}\right)$, $\left(\mathrm{NSGAI}I_{\mathrm{Min}\mathrm{Euclid}},\mathrm{FB}A_{Obj_{2}}\right)$, $\left(\mathrm{NSGAI}I_{\mathrm{Min}\mathrm{Euclid}},\mathrm{FB}A_{Obj_{3}}\right)$, the obtained *p*-values were 0.000018,0.000026, and 0.000087, respectively. These results mean a rejection of $H_{0}$, indicating a difference between the quality results of NSGAII and FBA, favoring NSGAII due to its lower values.

The results in Table 1 show that NSGAII improved FBA in terms of quality. Considering multiple objectives, NSGAII obatined closer solutions to the ideal point than those obtained by FBA. Moreover, the statistical test confirms that there is indeed a significant difference among these results. In addition, the good performance of NSGAII with respect to the diversity indicator D is also confirmed according to the values shown in Column 6, where the number of solutions ranges from a few dozens to several hundred, depending on the configuration, while classical FBA usually offers only one solution when Flux Variance Analysis (FVA) is not used.

**Figure 2:**
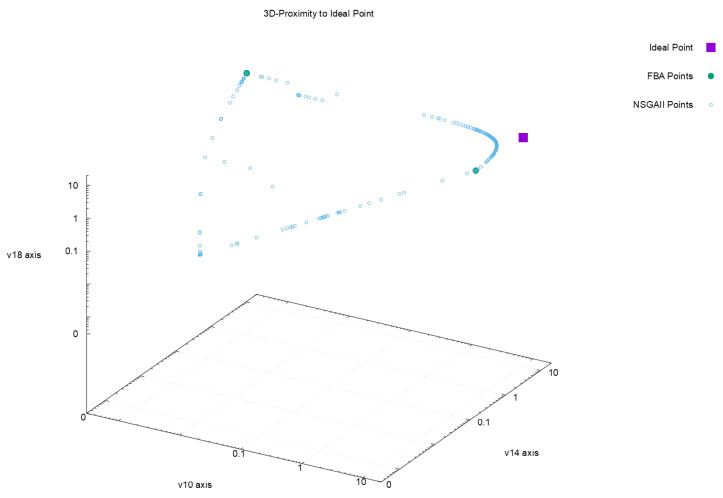
Pareto approximation for configuration $C_{0}$ with respect to the objectives $\left(v_{10},v_{14},v_{18}\right)$.

**Figure 3:**
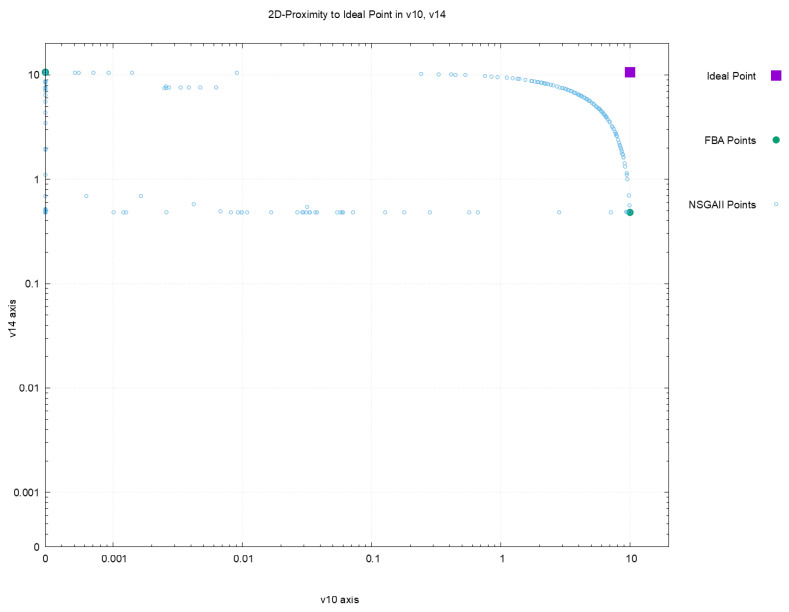
Pareto approximation for configuration $C_{0}$ with respect to the plane formed by objectives $\left(v_{10},v_{14}\right)$.

**Figure 4:**
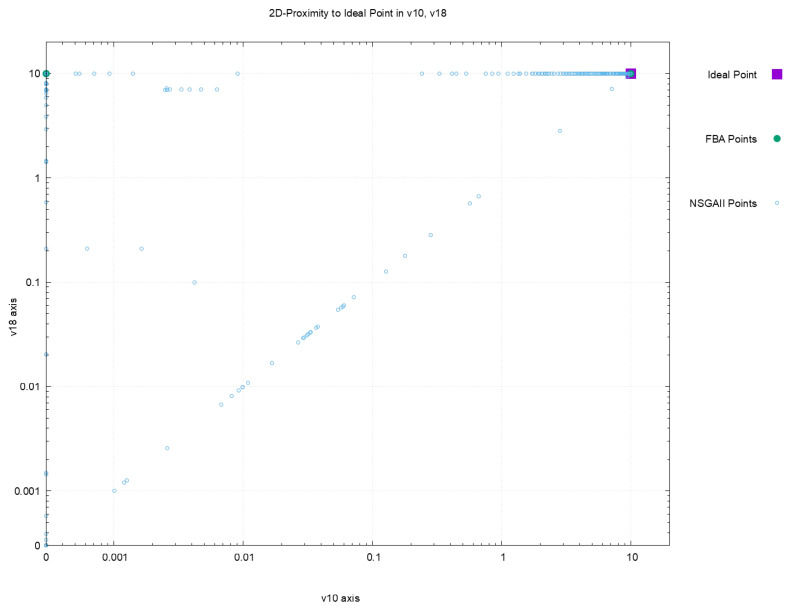
Pareto approximation for configuration $C_{0}$ with respect to the plane formed by objectives $\left(v_{10},v_{18}\right)$.

**Figure 5:**
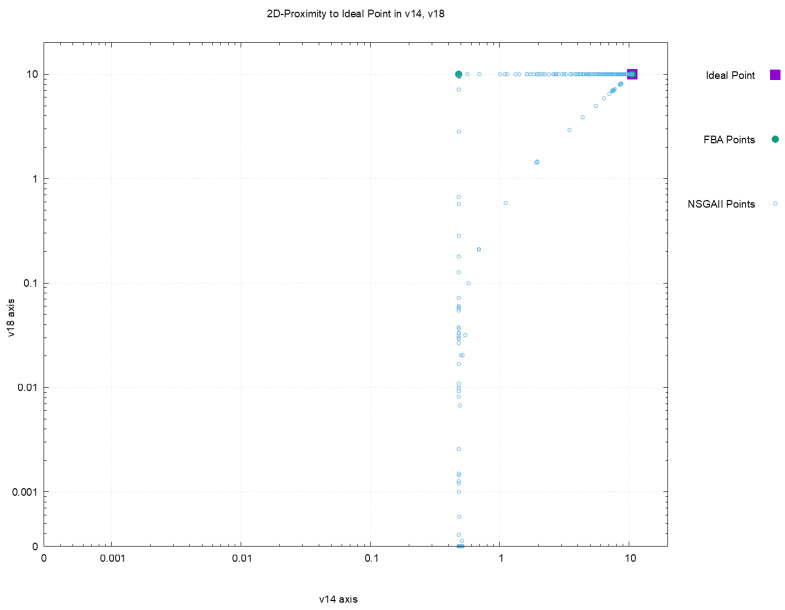
Pareto approximation for configuration $C_{0}$ with respect to the plane formed by objectives $\left(v_{14},v_{18}\right)$.

Figure 2, Figure 3, Figure 4 and Figure 5 offer a perspective of the behavior of NSGAII concerning the spread indicator S applied to the results achieved in the configuration $C_{0}$. All these figures show the ideal point in purple color, the three solutions reported by FBA in green color, and all the solutions reported by NSGAII are in blue circles. From these graphics, three main observations must be commented on: (1) first, the solutions of NSGAII describe the real contour of the Pareto frontier, while the solutions by FBA are only extreme points; (2) there exist solutions closers to the ideal point even though FBA warranty optimal solutions; and (3) the solutions of NSGAII are widely spread in the Pareto frontier. Hence, from the previous observations, it can be noted that NSGAII spread improves that of FBA; this behavior is repeated in all the remaining configurations.

A closer numerical look at the differences between NSGAII and FBA under configuration $C_{0}$ is shown in Table 2. This table compares the fluxes achieved by FBA in each case against some selected solutions reported by NSGAII.

Some additional insights arise from the previous results. Let us begin with the variety of configurations used in the comparison; this demonstrates the versatility of NSGAII to adapt to different circumstances and its capacity to improve the analysis of the metabolic network given the larger number of solutions produced for each of them.

After that, evolutionary approaches require fewer resources than FBA when dealing with multiple objectives; for example, it has the advantage of spending less time and memory. Approaches such as NSGAII allow a greater power of choice in the decision-making process also due to the variety and number of solutions, and the possibility of an easier recognition of the most important fluxes in a network and their influence and impact rather than not having a methodology.

Finally, by analyzing sets of several tens or hundreds of solutions simultaneously instead of just one through the classical approach, it is possible to have a better perspective of what is happening in cell metabolism.

## 3. Discussion

**Figure 6:**
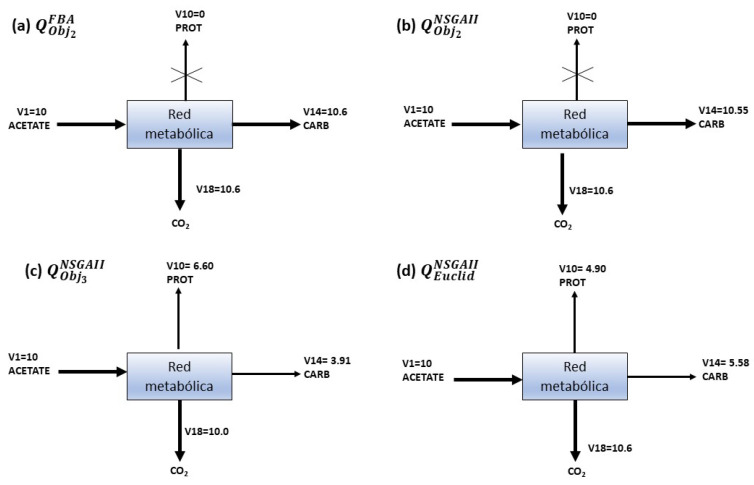
The distribution fluxes of objective function. Subfigure (**a**) shows the optimization of the $v_{14}$ flux using FBA; subfigures (**b**–**d**) correspond to different fluxes distributions obtained from NSGAII optimizing $v_{14}$, $v_{10}$ and $v_{18}$ simultaneously.

**Figure 7:**
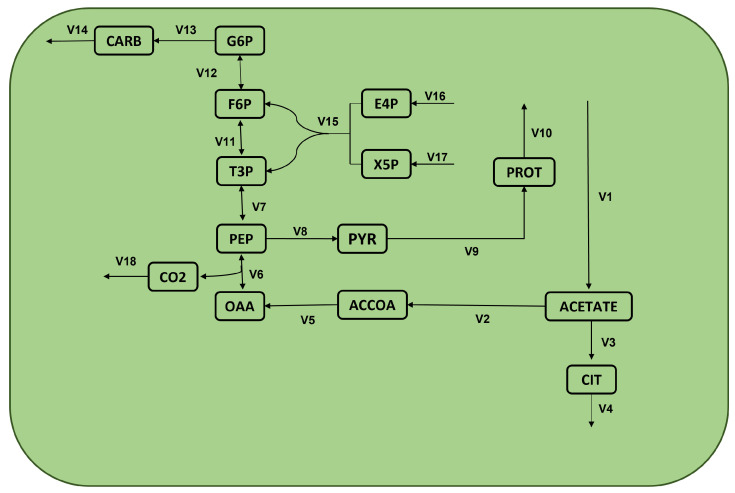
Metabolic network of *Chlamydomonas reinhardtii*.

Figure 6 is an oversimplifying of the *Chlamydomonas reinhardtii* metabolic network (detailed in Figure 7). The representation of Figure 6a are values obtained by optimizing the $v_{14}$ flux using FBA. Figure 6b–d correspond to results obtained from NSGAII. From these results, it can be seen that a cell always produces a compromise between the amount of biomass, carbon reserves, and its respiration process. Everything that occurs inside is distributed in distinct ways and proportions. The advantage of the proposed implementation of NSGAII is that one execution of the algorithm produces several fluxes distributions, in contrast with FBA which only produces one, the solutions’ set from NSGAII improve a researcher’s sight view of the physiological scenario under different conditions.

The growth on acetate as a carbon source of *Chlamydomonas reinhardtii* synthesizes CO${}_{2}$ as a product of metabolism, as seen in all fluxes distributions shown in Figure 6a–d, denoted $v_{18}$. In this case, the metabolism of carbon acts as a source of energy for biomass growth.

Figure 6c shows that the protein value of 6.60 is greater than the flux of carbohydrates that corresponds to 3.9, indicating a low accumulation of carbohydrates in favor of more significant protein production. This result concurs with the reported higher protein content observed when *Chlamydomonas reinhardtii* is cultured heterotrophically [15].

Figure 6d shows a slightly more uniform distribution of fluxes compared to the ones shown in Figure 6a, where all the fluxes are directed to the production of carbohydrates, compromising the entire flux of proteins $v_{10}$, leading to a state lacking growth in the biomass.

NSGAII can also produce solutions with similar fluxes’ values to those obtained from FBA. For example, in Figure 6b, values are almost equal to those in Figure 6a, where the produced fluxes point to the generation of Carbohydrates and CO${}_{2}$. However, these solutions compromise protein production, such situation implies null growth and an undesirable condition for a real process, as discussed in previous experimental research [15,37].

## 4. Materials and Methods

This section presents the proposed implementation for NSGAII, and the experiment conducted to validate it in the solution of MOFBA. It is organized into three subsections. First, it shows the proposed approach. After that, it presents the case study. Finally, it ends with the description of the experimental design used to test NSGAII.

### 4.1. Proposed Evolutionary Approach Based on NSGAII

Traditionally, the optimization of metabolic networks considers a steady-state approximation in which the metabolite concentrations are assumed to be constant. Since in the majority of the real-world metabolic networks the number of reactions (denoted by *n*) is higher than the number of metabolites (denoted by *m*), there is a large number of combinations of reaction fluxes that satisfy such systems. This section presents the proposed multi-objective FBA and a novel evolutionary approach that solves it, efficiently producing a proper approximation of the Pareto frontier with a quite diverse set of Pareto solutions that can improve decision-making in metabolic engineering.

The novel features included in this research are: (1) a novel encoding scheme for an FBA solution; (2) an original fitness evaluation based on classic FBA that guides the approximation of the Pareto front on MOFBA; and (3) a novel adaptation of the NSGAII framework to solve the particular MOFBA defined under the previous characteristics, so that feasible solutions can be achieved.

#### 4.1.1. Multi-Objective Optimization Model for FBA

Equation (1) shows the classic formulation for FBA. The solution of such an approach will yield a solution that maximizes the biomass. This work proposes the use of Equation (2) as the multi-objective alternative. It has a slight variation compared to Equation (1) which consists of the optimization of a set of bioproducts $\left\{v_{b_{1}},\cdots ,v_{b_{m}}\right\}$ instead of a single one. Equation (2) also considers the stoichiometric matrix *S*, the fluxes vector *v*, the steady-state condition Sv=0, and w.l.o.g. that all objectives are to be maximized.

(2) MOFBAmaxF(v)=(vb1,⋯,vbm)SubjecttoS·v=0LBj≤vj≤UBj,∀j∈{1,⋯,n}

MOFBA, as defined in Equation (2), cannot be solved using traditional linear solvers. Instead, enumerative schemes or approximated approaches must be used to achieve the Pareto frontier. The following section shows the novel evolutionary approach proposed in this work to solve this problem.

#### 4.1.2. Evolutionary Approach for MOFBA

This work proposes the use of a novel ensemble encoding the solution of MOFBA (as defined in Equation (2)) and its solution using NSGAII to solve the MOFBA. Given that NSGAII is an evolutive algorithm, it requires the definition of the following features: (1) encoding scheme; (2) fitness evaluation function; (3) genetic operators; (4) constraint handling strategy; and (5) population’s initialization method. The proposed ensemble of these components to handle MOFBA is detailed in the remainder of this section.

Proposed Encoding Scheme W. Let us consider a metabolic network $M_{N}$ constituted by a set of reactions V, and two subsets $V^{M},V^{b}\subseteq V$, where $V^{M}\cap V^{b}=\emptyset$, that represent the reactions of the metabolites of interest for a decision-maker. Moreover, let $v=(v_{1},\cdots ,v_{n})$ be the fluxes vector for V and let us assume that exist initial lower and upper bounds $LB_{i},UB_{i}$ for each $v_{i},1\leq i\leq n$. Then, the encoding scheme W proposes the redefinition of the bounds of each $v_{i}$ associated with a reaction in $V^{M}\cup V^{b}$ using two values $\left(I_{i},\Delta_{i}\right)$. The new bounds are computed as $LB_{i}^{new}=I_{i}$ and $UB_{i}^{new}=\left(UB_{i}-I_{i}\right)\Delta_{i}+I_{i}$. All the remaining fluxes will keep their bound values unchanged. In other words, the solution encodes changes in the bounds so that FBA solves $M_{N}$ using a prespecified bioproduct, which in this work is assumed to be $v_{1}^{b}$. The resulting encoding vector *w* is of size $2|V^{M}\left|+2\right|V^{b}|$. Table 3 shows a graphical example of *w* applied to a case with |V|=7, and $|V^{M}\left|=\right|V^{b}|=3$. Having as initial bounds $LB_{i}=0$, and $UB_{i}=10$, and considering $V^{M}=\left\{v_{1},v_{2},v_{3}\right\}$ and $V^{b}=\left\{v_{4},v_{5},v_{6}\right\}$, the use of *w* will results in the new bounds (LB,UB)={(5,7.5),(0,7.5),(2,8.4),(10,10),(2.5),(6.25),(7.5,8.75)}. Let us point out that any application of W over a reaction *i* uses its initial values for $LB_{i},UB_{i}$.

Fitness Evaluation Function (or FEF). Given that the information required on the bio-products is associated with specific reactions, the aptitude of a solution obtained by NSGAII on MOFBA is evaluated considering their fluxes values. Hence, the criteria or objective functions to be optimized will be the fluxes of the reactions corresponding to the chosen bioproducts of interest in $V^{b}$ and denoted $\left(v_{1}^{b},\ldots ,v_{m}^{b}\right)$. The proposed FEF requires a leading bioproduct. Then, having an encoded solution *w*, and taking as leading bioproduct $v_{1}^{b}$, FEF uses the redefined bound produced by the encoding scheme W and $v_{1}^{b}$ to create a single-objective metabolic model that can be solved by FBA. From the solution of the previous model, the corresponding flux values for $\left\{v_{1}^{b},\ldots ,v_{m}^{b}\right\}$ are taken as the objective values resulting from the encoding *w*.

Genetic Operators. These operators create new solutions by dynamically and randomly varying the values of decision variables on existing solutions. Their selection was due to their success in solving problems involving real-valued decision variables [38]. The chosen operators for mutation, crossover, and selection were Polynomial Mutation [39], SBXCrossover [40], and a simple yet reliable random selection, respectively.

Constraint Handling Scheme. The ensemble for NSGAII considers a feasibility constraint. This constraint is violated whenever FBA in the fitness function reports unfeasibility. The latter arises because the bounds defined by an encoded solution might cause no feasible solution exists. This work uses the constraint handling method proposed in [41] to overcome this situation. As the generations evolve in NSGAII, the competition among solutions will always prefer feasible solutions, despite the non-domination status. In the long run, such a strategy tends to eradicate unfeasible solutions at the final report of the algorithm.

Initialization method. A randomly generated initial population was considered as the input for NSGAII.

In general, the novel idea behind the previous ensemble is that NSGAII exerts selective pressure toward the Pareto frontier using the genetic operators, the ranking based on non-domination, the diversity control established by the crowding distance, and an adequate constraint handling strategy. The solutions, which indirectly define the flux values for vector *v* in FBA, will dynamically evolve in the algorithm, and those with better aptitude (i.e., that are feasible and improve the best the chosen bioproducts of the reactions) are kept for future generations. The final set of solutions provided by NSGAII represents a wider, more spread, and more promising combination of the reaction fluxes values that a decision maker can take as an advantage for their research.

The following section details the validation process for this proposed ensemble of NSGAII.

### 4.2. Case of Study: Metabolic Network of *Chlamydomonas reinhardtii*

This work analyzes the FBA of an essential module of the microalgae *Chlamydomonas reinhardtii*. Figure 7 presents the related metabolic network, and Table 4 the corresponding reactions. This case presents three bioproducts of interest: proteins, carbohydrates, and $CO_{2}$ (denoted here as $v_{10}$, $v_{14},$ and $v_{18}$). Moreover, these networks show three primary substrates: acetate, E4P, and X5P (or $v_{1}$, $v_{16},$ and $v_{17}$, respectively). Optimizing the three bioproducts $\left(v_{10},v_{14},v_{18}\right)$ having as control decision variables $\left(v_{1},v_{16},v_{17}\right)$ is the base to validate NSGAII. The next section presents specific details on the performed experiment.

### 4.3. Experimental Design

The experimental method evaluates the performance of NSGAII and compares it against the classical FBA. The hypothesis under consideration was that the decision-making on metabolic analysis can be improved using the evolutionary approach. The considered indicators that assess such improvement were the quality of the solutions, their diversity D, and their spread, or S concerning the Pareto frontier when considering the optimization of multiple fluxes. During the experiment, it was assumed that the study cases have the initial bounds limits in every reaction set to LB=0,UB=100.

Besides the set of bioproducts of interest, the configuration $C_{0}=\left\{v_{10},v_{14},v_{18}\right\}$, the experiment extends the analysis to the additional set of 36 configurations presented in Table 5. Each configuration represents a different set of bioproducts of interest. Demonstrating a good performance on such number of configurations would indicate that the evolutionary approach is versatile, and can easily be adapted to analyze metabolic networks under distinct contexts.

The quality of a solution, denoted Q, is measured as the Euclidean distance to an *Ideal Point*. Each configuration defines the ideal point and the best flux value resulting from FBA using each bioproduct of interest. For example, with the configuration $C_{0}$ the ideal point would be formed by the three optimal flux values reported by FBA, optimizing $v_{10}$, $v_{14}$, and $v_{18}$, respectively.

The diversity of solutions, or D, is assessed by the number of distinct solutions achieved by the evolutionary approach. Let us recall that FBA only provides an optimal solution. Finally, the spread or S will be graphically demonstrated by showing a broad distribution of solutions approximating the Pareto frontier.

The development of the proposed NSGAII for MOFBA was done by combining the FBA implementation from COBRAPY [42], and the NSGAII implementation from PyMETAL [43]. Both technologies were integrated into a Python script, the following NSGAII values as parameters: (1) the population size was set to N=100; (2) the stop criterion was defined as a maximum number of evaluations of 10,000; (3) for the *PolynomialMutation*, a mutation probability of 0.083 and a distribution index of 20 was set; and (4) for the *SBXCrossover* a crossover probability of 1.0 with a distribution index of 20 was set. Given the stochastic nature of NSGAII, each configuration was solved 31 times in order to have a representative sample of the solutions.

## 5. Conclusions

This study carried out the maximization of multi-objective functions of the production of proteins, carbohydrates, and $CO_{2}$ as a basis to demonstrate the use of multi-objective optimization with the NSGAII algorithm in a part of a reduced metabolic network of the microalga *Chlamydomonas reinhardtii*. The results obtained were compared with FBA analysis, achieving a similarity between the maximization of FBA when only a single objective function is maximized, but it also shows a broader perspective of the study of metabolic fluxes when there are multiple objective functions, NSGAII provide not only solutions closer to the ideal point in quality but also a better diversity and spread of them. Moreover, as far as we know, this is the first approach using evolutionary algorithms for metabolic analysis.

The use of the NSGAII algorithm to facilitate the understanding of the behavior of the metabolism when using multi-objective analysis must take into consideration that a possible decision-maker in the case study is interested in a broader set of combinations of fluxes in the reaction, by maximizing or minimizing different objective functions simultaneously, to explore a wide set of solutions of which those that may be more convenient for the investigation of a species are more easy to extract; in addition, we obtained a broader perspective of the study of metabolic fluxes by knowing how the different settings assigned.

For future work, the NSGAII evolutionary algorithm will be applied to a much larger microalgae metabolic network, using different configurations by maximizing different objective functions and comparing the results with FBA, observing the different solutions according to the requirements, and taking the most suitable solutions according to the needs of the model. In addition, the use of the NSGAII algorithm compensates production between the fluxes of the metabolites of interest and the fluxes of biomass production.

## Figures and Tables

**Table 1:** Report on best Euclidean distances to the ideal point.

	Euclidean Distance to Ideal Point				
**Config.**	$Q^{\mathrm{NSGAII}}$	$Q_{{Obj}_{1}}^{\mathrm{FBA}}$	$Q_{{Obj}_{2}}^{\mathrm{FBA}}$	$Q_{{Obj}_{3}}^{\mathrm{FBA}}$	$|F_{0}|$
$C_{0}$	7.16	10.12	10	10	349
$C_{1}$	8.07	10.12	10	10	158
$C_{2}$	11.56	14.23	14.14	14.14	2501
$C_{3}$	11.56	14.23	14.14	14.14	1701
$C_{4}$	7.12	10.12	10	10	217
$C_{5}$	8.34	10.12	10	10	359
$C_{6}$	8.19	14.23	10	10	617
$C_{7}$	10	10.12	14.14	10	53
$C_{8}$	10	10.12	14.14	10	68
$C_{9}$	10	14.27	10	10	147
$C_{10}$	8.25	14.31	10	10	397
$C_{11}$	8.24	14.31	10	10	279
$C_{12}$	7.13	10.12	10	10	218
$C_{13}$	0	0	0	0	125
$C_{14}$	8.16	10	10	14.14	1821
$C_{15}$	8.16	10	10	14.14	1646
$C_{16}$	0	0	0	0	189
$C_{17}$	0	0	0	0	216
$C_{18}$	9.98	10	10	10	160
$C_{19}$	0	0	0	0	88
$C_{20}$	0	0	0	0	171
$C_{21}$	8.20	10.06	10.06	10	202
$C_{22}$	7.13	10.12	10.12	10	325
$C_{23}$	7.13	10.12	10.12	10	465
$C_{24}$	0	0.06	0.06	0	81
$C_{25}$	0	0	0	0	125
$C_{26}$	8.17	10	10	14.14	1597
$C_{27}$	8.18	10	10	14.14	1199
$C_{28}$	0	0	0	0	175
$C_{29}$	0	0	0	0	280
$C_{30}$	0	0	0	0	391
$C_{31}$	8.27	10	10	10	276
$C_{32}$	7.17	10	10	10	261
$C_{33}$	0	0	0	0	122
$C_{34}$	0	0	0	0	55
$C_{35}$	0	0	0	0	90
$C_{36}$	0	0	0	0	25

**Table 2:** Results.

		$Q_{{Obj}_{1}}^{\mathrm{FBA}}$	$Q_{{Obj}_{2}}^{\mathrm{FBA}}$	$Q_{{Obj}_{3}}^{\mathrm{FBA}}$	$Q_{{Obj}_{1}}^{\mathrm{NSGAII}}$	$Q_{{Obj}_{2}}^{\mathrm{NSGAII}}$	$Q_{{Obj}_{3}}^{\mathrm{NSGAII}}$	$Q_{\mathrm{Euclid}}^{\mathrm{NSGAII}}$
BY OBJECTIVE	$v_{10}$	0	10	0	0	9.99	3.93	5.099
	$v_{14}$	10.12	0	10	10.11	0.045	6.68	5.019
	$v_{18}$	0	0	0	0	0	0	0
EUCLIDEAN		10.12	10	10	10.119	10	7.49	7.15
OBJECTIVE	$v_{10}$	10	0	10	10	$3.8\times 10^{-9}$	6.60	4.90
	$v_{14}$	0.48	10.6	0.6	0.48	10.55	3.91	5.58
	$v_{18}$	10	10	10	10	10	10	10
FLUXES	$v_{1}$	10	10	10	10	10	10	10
	$v_{2}$	10	10	10	10	10	10	10
	$v_{3}$	0	0	0	0	0	0	0
	$v_{4}$	0	0	0	0	0	0	0
	$v_{5}$	10	10	10	10	10	10	10
	$v_{6}$	10	10	10	10	10	10	10
	$v_{7}$	0	10	0	0	9.99	3.93	5.099
	$v_{8}$	10	0	10	10	$3.8\times 10^{-9}$	6.60	4.90
	$v_{9}$	10	0	10	10	$3.8\times 10^{-9}$	6.60	4.90
	$v_{10}$	10	0	10	10	$3.8\times 10^{-9}$	6.60	4.90
	$v_{11}$	0.24	10.3	0.3	0.24	10.27	3.65	5.34
	$v_{12}$	0.48	10.6	0.6	0.48	10.55	3.91	5.58
	$v_{13}$	0.48	10.6	0.6	0.48	10.55	3.91	5.58
	$v_{14}$	0.48	10.6	0.6	0.48	10.55	3.91	5.58
	$v_{15}$	0.24	0.3	0.3	0.24	0.27	0.26	0.24
	$v_{16}$	0.24	0.3	0.3	0.24	0.27	0.26	0.24
	$v_{17}$	0.24	0.3	0.3	0.24	0.27	0.26	0.24
	$v_{18}$	10	10	10	10	10	10	10

**Table 3:** Proposed solution’s encoding for MOFBA.

Encode Solution *w*											
Decision Variables						Objectives					
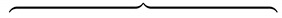						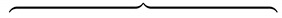					
**1**	**2**	**3**	**4**	**5**	**6**	**7**	**8**	**9**	**10**	**11**	**12**
$LB_{v_{1}^{M}}$	$\Delta_{v_{1}^{M}}$	$LB_{v_{2}^{M}}$	$\Delta_{v_{2}^{M}}$	$LB_{v_{3}^{M}}$	$\Delta_{v_{3}^{M}}$	$LB_{v_{1}^{b}}$	$\Delta_{v_{1}^{b}}$	$LB_{v_{2}^{b}}$	$\Delta_{v_{2}^{b}}$	$LB_{v_{3}^{b}}$	$\Delta_{v_{3}^{b}}$
5	0.50	0	0.75	2	0.80	10	1.00	2.5	0.50	7.5	0.10

**Table 4:** Reactions derived from the metabolic network Figure 7.

Name	Formula	Name	Formula
*v*_1_ :	–> acetate	*v*_10_ :	PROT –>
*v*_2_ :	acetate –> ACCOA	*v*_11_ :	T3P <=> F6P
*v*_3_ :	acetate –> CIT	*v*_12_ :	F6P <=> G6P
*v*_4_ :	CIT –>	*v*_13_ :	G6P –> CARB
*v*_5_ :	ACCOA –> OAA	*v*_14_ :	CARB –>
*v*_6_ :	OAA <=> PEP + CO_2_	*v*_15_ :	E4P + X5P –> F6P + T3P
*v*_7_ :	PEP <=> T3P	*v*_16_ :	–> E4P
*v*_8_ :	PEP –> PYR	*v*_17_ :	–> X5P
*v*_9_ :	PYR –> PROT	*v*_18_ :	CO_2_–>

**Table 5:** Experiment’s additional configurations of the reactions fluxes apart from $C_{0}=\left\{v_{10},v_{14},v_{18}\right\}$.

No.	Configuration	No.	Configuration	No.	Configuration
$C_{1}$	$\left\{v_{10},v_{14},v_{2}\right\}$	$C_{13}$	$\left\{v_{10},v_{18},v_{2}\right\}$	$C_{25}$	$\left\{v_{14},v_{18},v_{2}\right\}$
$C_{2}$	$\left\{v_{10},v_{14},v_{3}\right\}$	$C_{14}$	$\left\{v_{10},v_{18},v_{3}\right\}$	$C_{26}$	$\left\{v_{14},v_{18},v_{2}\right\}$
$C_{3}$	$\left\{v_{10},v_{14},v_{4}\right\}$	$C_{15}$	$\left\{v_{10},v_{18},v_{4}\right\}$	$C_{27}$	$\left\{v_{14},v_{18},v_{2}\right\}$
$C_{4}$	$\left\{v_{10},v_{14},v_{5}\right\}$	$C_{16}$	$\left\{v_{10},v_{18},v_{5}\right\}$	$C_{28}$	$\left\{v_{14},v_{18},v_{2}\right\}$
$C_{5}$	$\left\{v_{10},v_{14},v_{6}\right\}$	$C_{17}$	$\left\{v_{10},v_{18},v_{6}\right\}$	$C_{29}$	$\left\{v_{14},v_{18},v_{2}\right\}$
$C_{6}$	$\left\{v_{10},v_{14},v_{7}\right\}$	$C_{18}$	$\left\{v_{10},v_{18},v_{7}\right\}$	$C_{30}$	$\left\{v_{14},v_{18},v_{2}\right\}$
$C_{7}$	$\left\{v_{10},v_{14},v_{8}\right\}$	$C_{19}$	$\left\{v_{10},v_{18},v_{8}\right\}$	$C_{31}$	$\left\{v_{14},v_{18},v_{2}\right\}$
$C_{8}$	$\left\{v_{10},v_{14},v_{9}\right\}$	$C_{20}$	$\left\{v_{10},v_{18},v_{9}\right\}$	$C_{32}$	$\left\{v_{14},v_{18},v_{2}\right\}$
$C_{9}$	$\left\{v_{10},v_{14},v_{11}\right\}$	$C_{21}$	$\left\{v_{10},v_{18},v_{11}\right\}$	$C_{33}$	$\left\{v_{14},v_{18},v_{2}\right\}$
$C_{10}$	$\left\{v_{10},v_{14},v_{12}\right\}$	$C_{22}$	$\left\{v_{10},v_{18},v_{12}\right\}$	$C_{34}$	$\left\{v_{14},v_{18},v_{2}\right\}$
$C_{11}$	$\left\{v_{10},v_{14},v_{13}\right\}$	$C_{23}$	$\left\{v_{10},v_{18},v_{13}\right\}$	$C_{35}$	$\left\{v_{14},v_{18},v_{2}\right\}$
$C_{12}$	$\left\{v_{10},v_{14},v_{15}\right\}$	$C_{24}$	$\left\{v_{10},v_{18},v_{15}\right\}$	$C_{36}$	$\left\{v_{14},v_{18},v_{2}\right\}$

## Data Availability

The data presented in the study is available in the article.

## Abbreviations

The following abbreviations are used in this manuscript:

ACCOA	Acetyl-coenzyme A
CIT	Citrato
OAA	Oxaloacetate
PEP	Phosphoenol pyruvate
T3P	Dihydroxyacetone phosphate and 3-phosphoglycerate
PYR	Pyruvate
PROT	Protein
F6P	Fructose-6-phosphate
G6P	Glucose-6-phosphate
CARB	Carbohydrates
FBA	Fluxes balance analysis
CO_2_	Carbon dioxide
NSGAII	Genetic Algorithm of Non-Dominated Classification
MO-FBA	Multi-objective flux balance analysis
MO-FVA	Multi-objective flux variability analysis
MOEAs	Multi-objective Evolutionary Algorithms
SBX	Simulated Binary Crossover
